# Cheating, facilitation and cooperation regulate the effectiveness of phage‐encoded exotoxins as antipredator molecules

**DOI:** 10.1002/mbo3.636

**Published:** 2018-04-19

**Authors:** Iqbal Aijaz, Gerald B. Koudelka

**Affiliations:** ^1^ Department of Biological Sciences University at Buffalo Buffalo NY USA

**Keywords:** Bacteria, bacteriophage, cooperation, horizontal gene transfer, phagocytosis, public good, *Tetrahymena*

## Abstract

Temperate phage encoded Shiga toxin (Stx) kills the bacterivorous predator, *Tetrahymena thermophila*, providing Stx^+^ *Escherichia coli* with a survival advantage over Stx^−^ cells. Although bacterial death accompanies Stx release, since bacteria grow clonally the fitness benefits of predator killing accrue to the kin of the sacrificed organism, meaning Stx‐mediated protist killing is a form of self‐destructive cooperation. We show here that the fitness benefits of Stx production are not restricted to the kin of the phage‐encoding bacteria. Instead, nearby “free loading” bacteria, irrespective of their genotype, also reap the benefit of Stx‐mediated predator killing. This finding indicates that the phage‐borne Stx exotoxin behaves as a public good. Stx is encoded by a mobile phage. We find that Stx‐encoding phage can use susceptible bacteria in the population as surrogates to enhance toxin and phage production. Moreover, our findings also demonstrate that engulfment and concentration of Stx‐encoding and susceptible Stx^−^ bacteria in the *Tetrahymena* phagosome enhances the transfer of Stx‐encoding temperate phage from the host to the susceptible bacteria. This transfer increases the population of cooperating bacteria within the community. Since these bacteria now encode Stx, the predation‐stimulated increase in phage transfer increases the population of toxin encoding bacteria in the environment.

## 1 INTRODUCTION

Cooperation between individual cells is a widespread phenomenon occurring both within multicellular organisms and among individual microbes in a population. Cooperation among microbes takes many forms, including the production of public goods, that is, diffusible chemical resources that provide fitness benefit(s) to all nearby cells, regardless of kinship.

In nature, predation is responsible for about half of bacterial mortality (Brussow, Canchaya, & Hardt, 2004). As such, bacteria in all phyla have developed an array of antipredator defense strategies. Our earlier investigations demonstrate that Shiga toxin (Stx) encoding bacteriophage in *E. coli* can provide a bacterial population with the ability to combat predation by phagocytic predators (Arnold & Koudelka, 2014; Lainhart, Stolfa, & Koudelka, 2009; Mauro, Opalko, Lindsay, Colon, & Koudelka, 2013; Stolfa & Koudelka, 2012). We also showed previously that in the face of attack by predator, a only minor subset of bacteria lysogenized with temperate Stx‐encoding lambdoid phage produce and release sufficient exotoxin to substantially reduce of predation the balance of the (Arnold & Koudelka, 2014; Lainhart et al., 2009; Stolfa & Koudelka, 2012). This finding suggests that Stx acts as a public good.

In cooperation mediated by public goods, producer cells both benefit from and bear the costs of production. Since exotoxins encoded by temperate lambdoid phages are produced only during lytic growth and their release depends on phage genes that induce bacterial cell lysis (Arnold & Koudelka, 2014; Lainhart et al., 2009), bacterial death accompanies exotoxin release. Therefore, similar to other examples of public goods‐mediated bacterial cooperation (Paton, 1996; Voth & Ballard, 2005), Stx‐mediated protist killing is a form of altruism known as self‐destructive cooperation (Ackermann et al., 2008). Since bacteria grow clonally, in the case of Stx‐mediated predator killing, the benefits accrue to the kin of the altruistic organism. However, since Stx is a diffusible public good that theoretically could benefit all potential protist prey, this cooperative behavior may be susceptible to ‘cheating’ in the face of selection pressure. Cheaters can reap the benefits of cooperation without bearing the costs of production. In the extreme, the benefits of the public good can accrue primarily to cells other than the producer. Since many bacterial predators do not discriminate between bacterial food sources, exotoxin‐mediated predator killing may enhance survival of nearby, but unrelated, ‘cheater’ bacteria. It is unknown whether this is the case with Stx‐mediated cooperation.

Cheating creates a dilemma for a population. If the cost of production exceeds the direct benefit to the producer, and cheaters benefit, but do not pay the cost of secretion, then they are expected to outcompete cooperating producers. Thus, without a mechanism to support cooperation, the fraction of cells producing the public good will decline in frequency resulting in the loss of the public good, a condition which would decrease the fitness of the entire population, producers and cheaters alike (Nowak, 2006).

Cooperation can be maintained in the face of cheating if specific mechanisms to allow preferential utilization of public goods by cooperating cells arise in the population. For example, cooperation is favored by the physical association of cooperative partners or if there is limited diffusion of the public good away from the cooperating producers (Brown & Buckling, 2008; Kummerli, Griffin, West, Buckling, & Harrison, 2009). This observation is consistent with kin selection theory, which postulates that cooperative interactions will evolve and be maintained if the benefits preferentially accrue to organisms that carry cooperation genes (Hamilton, 1964). Transmission of cooperation genes to nonproducers can also support cooperation by increasing genetic similarity. The increased genetic‐relatedness of the individuals in the population increases both the direct and indirect benefit of public goods transactions (Gardner, West, & Wild, 2011; West, Griffin, Gardner, & Diggle, 2006).

Strikingly, genes encoding cooperative traits are often found associated with mobile genetic elements such as plasmids or transposons (Dimitriu et al., 2014). These mobile elements facilitate rapid horizontal gene transfer within and between bacterial lineages suggesting that they can quickly alter the cooperative social structure of a population. Although the spread of plasmid‐encoded antibiotics resistance factors serves as an important and well‐studied case of horizontal gene transfer (HGT) mediated stabilization of public goods cooperation (Yurtsev, Chao, Datta, Artemova, & Gore, 2013), the ability of temperate bacteriophage to serve as a conduit for the horizontally transfer of public goods genes has not been thoroughly explored. The genomes of temperate prophage are found at surprisingly high frequency within the chromosomes of these organisms. The evolution of phage resistance occurs relatively easily. Thus, since the ultimate goal of bacteriophage is to reproduce, and in doing so, these phages kill their host, the prevalence of phage DNA, inside host chromosomes suggests that phage are tolerated because their presence provides an evolutionary advantage to the host.

While Stx release enhances the survival of the kin of Stx‐encoding bacteria in the presence of bacterial (Arnold & Koudelka, 2014; Lainhart et al., 2009; Stolfa & Koudelka, 2012), it is unknown whether the fitness benefits of Stx antipredator activity extend to the unrelated bacteria in the population. Also, since Stx is encoded on a mobile bacteriophage, the movement of this phage between bacteria could greatly impact the social structure of the bacterial population. That is, predation could encourage the transfer of phage to susceptible bacteria. Such transfers could lead to exploitation of these bacteria by using them to enhance public goods production and/or by creating new lysogenic cooperating bacterial. To test these ideas, we determined if phage‐mediated dissemination of a public good impacts the population of cooperating producers and cheaters in the face of predation. Given the contribution of Stx and other temperate lambdoid phage‐encoded exotoxins to serious human disease, this information may aid in understanding the increasing occurrence and virulence of these diseases.

## 2 EXPERIMENTAL PROCEDURES

### 2.1 Strains

EDL933 and MG1655were obtained from the ATCC. EDL933Δ*stx*, an EDL933 variant bearing deletions of all *stx* genes (Gobert et al., 2007) was obtained from Christine Miller, Institut National de la Recherche Argonomique. MG1655^λimm933W^ was obtained as a gift from David Friedman, University of Michigan (Tyler, Mills, & Friedman, 2004). MG1655^r*ecA*^ and EDL933^r*ecA*^ were created as described earlier (Shkilnyj & Koudelka, 2007). *T. thermophila* strains CU427.4 and A1868, were obtained from the Tetrahymena Stock Center (Cornell University). Antibiotics were added in the following concentration; ampicillin 100 μg/ml, chloramphenicol 50 μg/ml and tetracycline 25 μg/ml. All DNA oligonucleotide primers and *Taq*Man probes were purchased from IDT Technologies (Coralville, IA).

### 2.2 Construction of antibiotic resistant strains

MG1655 and MG1655^λimm933^were transformed with pET‐17b vector to generate MG1655(AMP) and MG1655^λimm933^(AMP). MG1655^r*ecA*^ (CAT, TET) was made by transforming a vector pACYC182 harboring tetracycline resistance gene into MG1655^r*ecA*^.2

### 2.3 Preparation of *Tetrahymena* and bacteria

Bacteria and *Tetrahymena* cells were prepared as follows: Cultures of the specified bacteria (Stx^+^: EDL933, Stx^−^: EDL933ΔStx, immune strain: MG1655^λimm933^,phage‐ susceptible strain: MG1655::AMP) were grown to saturation at 37°C in M9 plus 0.08% glucose supplemented with antibiotics when appropriate. The cultures were centrifuged at 8,000*g* for 10 min, washed twice with M9 plus 0.08% sodium citrate, and resuspended in M9 plus 0.08% sodium citrate. *Tetrahymena* cells were diluted fivefold from saturated liquid cultures and grown for 3 days in proteose peptone plus FeCl_2_ at 30°C. The cells were centrifuged at 5000*g* for 5 min, washed three times with 10 mmol/L Tris‐HCl (pH 7.4), and suspended in M9 plus 0.08% sodium citrate in a volume sufficient to give 10^4^ cells/ml. To each washed *Tetrahymena* culture, 10^8 ^cells/ml of the indicated bacteria were added and the cocultures were maintained at 30°C.

### 2.4 *Tetrahymena* and bacterial viability in mixed cocultures

To create artificial microcosms, we cocultured the indicated bacterial strains without or with *Tetrahymena*. In all cases, the total number of bacterial cells in these microcosms was 10^8 ^cells/ml. When present, *Tetrahymena* were added at 10^4 ^cells/ml. In microcosms in which the ratio of toxin and nontoxin producing bacteria was varied, the total number of bacteria was maintained at 10^8 ^cells/ml, only the ratio of toxin producing to nontoxin producing bacteria was altered. The microcosms were maintained by shaking at 30°C. At *t *=* *0, and 6 hr, two aliquots were removed from these microcosms. One aliquot was used to determine the change in *Tetrahymena* cell count. The cell count was obtained by counting the number of Lugol stained cells, visualized in a hemocytometer (Lainhart et al., 2009). *Tetrahymena* cells killed by exposure to Stx‐expressing bacteria are not visible, presumably because they have lysed. The second aliquot was used to determine the number of bacteria by plating the dilutions of cultures on agar plates containing 100 μg/ml ampicillin and determining the number of colony forming units (CFU). Each measurement was performed in duplicate and the data were averaged. Each experiment was repeated a minimum of three times. The data presented are the average of the three (or more) replicates.

### 2.5 Quantification of Stx^+^ phage 933W via Real‐Time qPCR

Bacteria and *Tetrahymena* were cocultured at 30°C for 4 hr as described above. Aliquots were taken at *t *=* *0, and 4 hr, and total DNA was extracted using InstaGene matrix (Biorad, Hercules CA) following the manufacturer's instructions. The primers and probes targeting *stx2*A (*stx2* ^*+*^ phage specific) gene were;

Forward primer 5′‐ATTAACCACACCCCACCG‐3′,

Reverse primer 5′‐GTCATGGAAACCGTTGTCAC‐3′,

*Taq*Man probe 5′‐CAGTTATTTTGCTGTGGATATACGA‐3′ labeled with fluorescent reporter dye HEX at the 5′ end and with the Black Hole Quencher (BHQ_1) at the 3′ end (Mauro et al., 2013).The primers and probes targeting the *uidA* (*E. coli*) gene were,

Forward primer 5′‐GTGTGATATCTACCCGCTTCGC‐3′,

Reverse primer 5′‐ AGAACGGTTTGTGGTTAATCAGGA‐3′, and

*Taq*Man probe 5′‐ TCGGCATCC GGTCAGTGGCAG T‐3′ which was labeled with the fluorescent reporter dye FAM (6‐carboxyfluorescein) at the 5′ end and with BHQ_1 at the 3′ end (Mauro et al., 2013).

The *stx2*A and *uidA* genes were amplified as a 20 μl reaction mixture using the PCR MasterMix in Bio‐Rad iQ5 real‐time PCR detection system. The following two‐step thermal profile was used: 5 min at 95°C, 45 repeats of 10 s at 95°C, 45 s at 60°C. Standard curves for the real‐time PCR analysis were made using genomic DNA containing the regions of interest.

### 2.6 Detection and quantification of new prophage formation

*Stx* ^*+*^phage donor (EDL933) and phage susceptible *E. coli* strain (MG1655) were coincubated in a ratio of 1:25, respectively, in the absence or presence of *Tetrahymena*. The microcosms were maintained at 30°C for 12 hr. *Tetrahymena* were separated from bacteria by differential centrifugation at 1,000*g* for 2 min, washed 5× with10 mmol/L Tris‐HCl (pH 7.4), lysed (Bolivar & Guiard‐Maffia, 1989), and internalized bacteria were diluted 1:200 fold in LB supplemented with 50 μg/ml chloramphenicol and 25 μg/ml tetracycline. Cells were grown to saturation at 30°C. DNA was extracted using InstaGene matrix, *stx2*A_*,*_ *uidA*, and *rbfE* genes were amplified using the following thermal profile: 5 min at 95°C, 30 repeats of 10 s at 95°C, 30 s at 60°C, 30 s at 72°C, and 5 min elongation at 72°C. The same primers were used to amplify *stx2A*, and *uidA* as mentioned above whereas the following primers were used to amplify *rbfE*:

### 2.7 Forward primer 5′‐CTACAGGTGAAGGTGGAATGGT‐3′

Reverse primer 5′‐GTAGCCTATAACGTCATGCCAAT‐3′ (Desmarchelier et al., 1998). The PCR products were separated on a 5% native PAGE gel. To measure the frequency of lysogenization, the microcosms containing EDL933, MG1655^*recA* (CAT, TET)^, and *Tetrahymena* were maintained at 30°C for 24 hr. At *t *=* *12, and 24 hr, *Tetrahymena* cells were separated from bacteria by differential centrifugation at 1,000*g* for 2 min, washed 5× with10 mmol/L Tris‐HCl (pH 7.4), lysed (Bolivar & Guiard‐Maffia, 1989), and internalized bacteria were diluted 1:200 fold in LB supplemented with 50 μg/ml chloramphenicol and 25 μg/ml tetracycline. Cells were grown to saturation at 30°C, DNA extracted, and *stx2a* and *uidA* genes were amplified as a 20 μl reaction mixture using the PCR MasterMix in Bio‐Rad iQ5 real‐time PCR detection system. Control experiments established that the amplification efficiency of the *stx2A* and *uidA* genes are identical under our conditions. The *stx2A*:*uidA* ratio represents the fraction of MG1655^*recA* (CAT TET)^ that had *stx2* ^*+*^ prophage. The number of lysogens in the population of MG1655^*recA* (CAT, TET)^ was calculated by multiplying the ratio of *stx2A*:*uidA* with total number of bacteria.

### 2.8 Statistical methods

Error bars presented in the figures represent standard deviations of the means of multiple (≥3) replicate experiments. *t* test was used to test the significance of differences between the mean of the measured initial amounts and the amounts of bacteria and/or *Tetrahymena* after treatment in each experiment.

## 3 RESULTS

### 3.1 Stx as an antipredator public good

**Figure 1:**
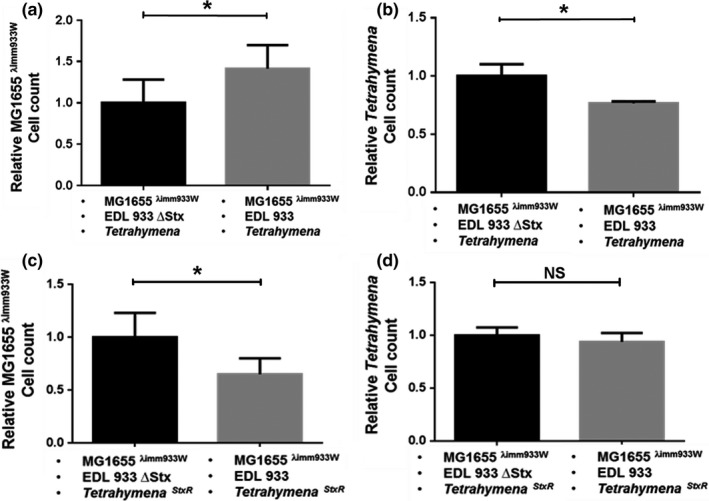
Effect of Stx encoding bacteria on the populations of *Tetrahymena thermophila* and phage‐immune (MG1655^λimm933W^) bacteria in artificial microcosms. Cells were prepared as described in Experimental Procedures. The microcosms contain EDL933*Δstx* (*Stx* ^*−*^) (black bars) or EDL933 (*Stx* ^*+*^) (gray bars) nontoxin producing Stx2‐phage immune MG1655^λimm933W^ and either Stx‐sensitive (a,b) or resistant (c,d) *T. thermophila*. The relative cell count of MG1655^λimm933W^ (a,c), and *Tetrahymena* (b,d) after 6 hr of co‐incubation are reported. Data are presented as the amount of MG1655^λimm933W^ bacteria or *Tetrahymena* cells surviving 6 hr co‐incubation relative to the number of cells at *t* = 0. Cell counts were obtained as described in Experimental Procedures. Error bars represent standard deviations from ≥3 independent experiments, *indicates *p *<* *.05. NS *p *>* *.05

To verify that Stx can act as a public good, we examined the effect of EDL933, a *stx* ^*+*^ phage‐bearing Shiga toxin encoding *E. coli* (STEC) bacterial strain or its *stx* ^−^ derivative, EDL933Δ*stx*, on the survivorship of MG1655^λimm933^, an *stx* ^−^ *E. coli* strain that cannot be infected by *stx2* ^*+*^ phage, in cocultures without or with predatory *Tetrahymena*. The EDL933 and MG1655^λimm933^ strains were distinguished by marking them with different antibiotic resistance genes. However, in cocultures containing *Tetrahymena*, the presence of the Stx‐encoding EDL933 strain increased the survival of the nontoxin‐encoding MG1655^λimm933^ strain 1.5‐fold more than cocultures containing EDL933Δ*stx* (Figure 1a). These observations indicate that the benefits of Shiga toxin's antipredator activity accrue to both the toxin producing and nontoxin‐producing cells in the bacterial population. These findings are consistent with the idea that Stx functions as a public good. These results also indicate that nontoxin‐producing cells in the bacterial population can cheat, that is, take advantage of the fitness benefits of Stx, without paying the fitness costs of its production.

To verify that the observed survival enhancement of MG1655^λimm933^ seen in Figure 1a is a consequence of the anti‐predator activity of Stx released by EDL933, we examined the growth of *Tetrahymena* in these cocultures. We found that, as compared to *Tetrahymena*‐containing cocultures with MG1655^λimm933^ and EDL933Δ*stx*, the amount of *Tetrahymena* decreased in cocultures containing EDL933 and MG1655^λimm933^, (Figure 1b). We also examined the survival of MG1655^λimm933^ in cocultures with EDL993 or EDL933Δ*stx* and a *Tetrahymena* strain that is resistant to Stx cytotoxic effects. Consistent with the phenotype of this *Tetrahymena* strain, the survival of the Stx‐resistant *Tetrahymena* strain was identical in cocultures containing either EDL933 or EDL933Δ*stx* (Figure 1d). Surprisingly the survival of MG1655^λimm933^ is decreased in cocultures containing EDL933 (Figure 1c). Regardless, the Stx‐mediated survival advantage of Stx2‐phage immune nontoxin‐encoding MG1655^λimm933^ is seen only under conditions where Stx acts as an antipredator molecule.

### 3.2 Role of Stx phage infection in regulating bacterial survival during predation

**Figure 2:**
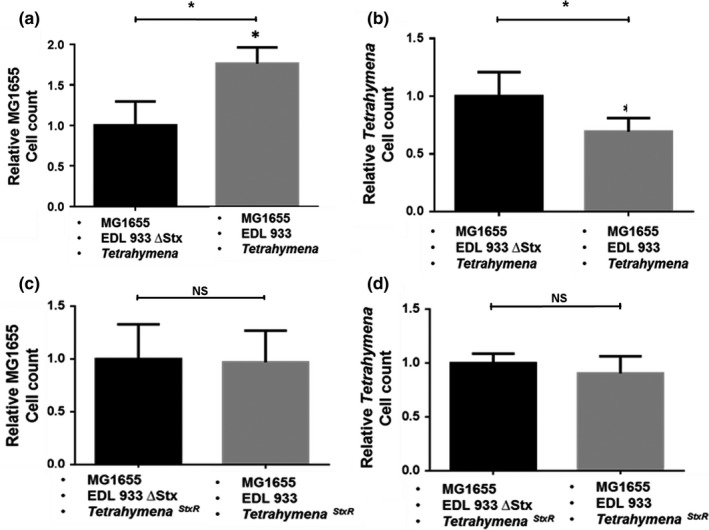
Effect of Stx encoding bacteria on the populations of *T. thermophila* and phage susceptible (MG1655) bacteria in artificial microcosms. Cells were prepared as described in Experimental Procedures. The microcosms contain EDL933*Δstx* (*Stx* ^*−*^) (black bars) or EDL933 (*Stx* ^*+*^) (gray bars) nontoxin producing phage susceptible MG1655 and either Stx‐sensitive (a,b) or resistant (c,d) *T. thermophila*. The relative cell count of MG1655 (a,c), and *Tetrahymena* (b,d) after 6 hr of coincubation are reported. Data are presented as the amount of MG1655 bacteria or *Tetrahymena* cells surviving 6 hr co‐incubation relative to the number of cells at *t* = 0. Cell counts were obtained as described in Experimental Procedures. Error bars represent standard deviations from ≥ 3 independent experiments, * indicates *p *<* *.05. NS *p *>* *.05

To probe the effect of phage infection on bacteria survival and predator killing, we repeated the above experiment, but replaced the Stx2‐phage resistant nontoxin‐encoding MG1655^λimm933^ strain with the *stx* ^*+*^ phage‐susceptible nontoxin‐encoding *E. coli* strain, MG1655. In these experiments, we find that in presence of *Tetrahymena*, the number of MG1655 cells that survive in cocultures containing EDL933 increases ~1.75‐fold over that in cocultures containing EDL933Δ*stx* (Figure 2a). The results also indicate that the Stx‐dependent survival enhancement of phage‐susceptible MG1655 is greater than that seen with the phage‐immune MG1655^λimm933^. This observation suggests that MG1655 may be used as surrogate for phage growth and Stx production (see below). Consistent with this suggestion, control experiments confirm that in the absence of *Tetrahymena*, the amount of MG1655 is identical in cocultures with either EDL993 or EDL933Δ*stx*, showing that the increased bacterial survival of MG1655 in the presence of *Tetrahymena* is a consequence of a higher level of predator killing.

Similar to what we observe with MG1655^λimm933^, an Stx‐dependent increase in the survival of nontoxin‐encoding MG1655 is also seen in the presence of predatory, Stx susceptible *Tetrahymena* (Figure 2a). The survival increase under these conditions is a consequence of the Stx‐dependent reduction in the survival of *Tetrahymena* (Figure 2b). In contrast the survival advantage of nontoxin‐encoding MG1655 is not seen in the presence of Stx‐resistant *Tetrahymena*, (Figure 2c,d). Taken together, the results in Figures 1, 2 indicate the Shiga toxin's antipredator activity benefits the survival of bacteria that are not direct kin of the Stx producing bacteria. This observation indicates that phage‐encoded Stx acts as a public good. These results also show that phage‐resistant, nontoxin‐encoding MG1655^λimm933^ can ‘cheat’, taking advantage of the antipredator activity of Stx released by EDL933, while not investing in its production.

### 3.3 Impact of phage‐susceptible and phage‐immune bacterial strains on the predation resistance of bacterial populations

The placement of Stx on a mobile phage provides a method for both increasing production of this public good and amplifying the allele that encodes this product. That is, the presence of phage‐susceptible strains in the bacterial population may be used as ‘surrogates’ to amplify both phage and toxin. This scenario would lead to increased predator death and thereby increased bacterial survival. To test this idea and to determine the impact of population structure on its resistance to protist predation, we measured bacterial and *Tetrahymena* survival in cocultures containing *Tetrahymena*, EDL933 (phage^+^/Stx2^+^) or EDL933Δ*stx* (phage^+^/Stx^−^) and varying ratios of bacteria that are either susceptible (MG1655) or immune (MG1655^λimm933W^) to infection by the *stx2* phage in EDL933.

**Figure 3:**
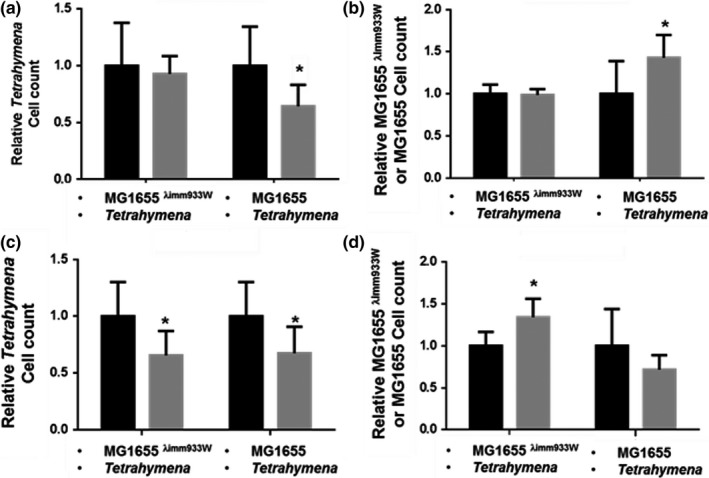
Effect of Stx‐encoding bacteria on the population of *T. thermophila* and nontoxin‐producing bacteria in artificial microcosms having varying ratios of phage immune or phage‐susceptible to toxin producing (*Stx* ^*+*^) and nontoxin producing (*Stx* ^*−*^) bacteria. Data are presented as the relative change in the amount of MG1655 (susceptible) or MG1655^λimm933W^ (immune) bacteria (a,c) and *Tetrahymena* (b&d) cells surviving 6 hr coincubation relative to the number of cells at *t* = 0. Microcosms in a,b contained *Tetrahymena*, phage^+^/Stx^−^ EDL933Δ*stx* (solid black/black striped bars) or phage^+^/Stx^+^ EDL933 (solid gray/gray hatched bars), and a 10‐fold excess of phage immune MG1655^λimm933W^ (hatched/striped bars) or phage susceptible MG1655 (solid bars). Microcosms in c,d consisted of *Tetrahymena*, 10‐fold excess phage^+^/Stx^−^ EDL933Δ*stx* (solid black/black striped bars) or EDL933 (solid gray/gray hatched bars) over phage immune MG1655^λimm933W^ (hatched/striped bars) or phage susceptible MG1655, (solid bars). Cell counts were obtained as described in Experimental Procedures. Error bars represent standard deviations from ≥3 independent experiments, * indicates *p *<* *0.05. NS *p *>* *0.05

In cocultures containing *Tetrahymena*, EDL933 (phage^+^/Stx2^+^), and the phage susceptible MG1655 *E. coli* strain present in 10‐fold excess over EDL933, the survival of MG1655 increases 1.5‐fold (Figure 3a, gray solid bars), and the number of surviving *Tetrahymena* decreases (Figure 3b, gray solid bars), relative to cocultures in which EDL933Δ*stx* (phage^+^/Stx2^−^) is used in place of EDL933 (Figure 3a,b black solid bars). In cocultures containing *Tetrahymena* and a 10‐fold excess of ‘immune’ MG1655^λimm933W^ *E. coli,* the survival of the immune strain is identical regardless of whether the coculture also contains EDL933 or EDL933Δ*stx* (Figure 3a, patterned bars). The number of surviving *Tetrahymena* is also identical in these two coculture conditions (Figure 3b, patterned bars).

MG1655^λimm933^ cannot be infected by Stx‐encoding phage released from EDL933. Hence, the findings in Figure 3a,b suggest that in cocultures containing a smaller amount of EDL933 and a larger amount of MG1655^λimm933W^, the amount of toxin produced is apparently too low to affect predator viability. On the contrary, in cocultures containing a smaller amount of EDL933 and a larger amount of ‘susceptible’ *E. coli* MG1655, the predator viability decreased, suggesting that the *stx* ^*+*^ phage released by EDL933 is using phage susceptible *E. coli* as a surrogate to amplify the amount of exotoxin produced by the population.

Consistent with this idea, in cocultures containing *Tetrahymena* and EDL933 (phage^+^/Stx2^+^) in 10‐fold excess over the immune MG1655^λimm933W^, the amount of surviving *Tetrahymena* decreased, relative to similar cocultures containing EDL933Δ*stx* (phage^+^/Stx2^−^) instead of EDL933 (Figure 3d patterned bars). Under these conditions, the number of surviving of MG1655^λimm933W^ in the EDL933‐containing cocultures increases 1.3‐fold than those containing EDL933Δ*stx* (Figure 3c patterned bars). Similarly, in cocultures containing *Tetrahymena* and a 10‐fold excess of EDL933 over phage susceptible MG1655, the amount of surviving *Tetrahymena* also decreased as compared to cocultures containing EDL933Δ*stx* (Figure 3d solid bars). In these cases, since the toxin‐producing EDL933 strain is in excess, the amount of Stx produced by this strain alone is sufficient to kill the predator and allow either of the nontoxin‐encoding bacteria in the population to benefit from the antipredator activity of Stx.

Interestingly, in these cocultures, the number of surviving MG1655 does not increase (Figure 2c solid bars), as would be expected from Stx‐mediated killing of predatory *Tetrahymena*. We hypothesize that the inability of MG1655 to increase its population size in the face of lower predator numbers may be a consequence of phage‐mediated lysis of this strain in these cocultures (see below and Discussion).

In addition to showing that the ability of exotoxins to kill bacterivorous predators is exploited by unrelated bacterial ‘cheaters’, the results in Figure 3 indicate that MG1655 can be used as a surrogate, to amplify the amount of exotoxin produced by the population and facilitate enhanced population survival by killing predatory *Tetrahymena*. We wished to confirm that the enhanced killing is a consequence of lytic growth of Stx‐encoding phage released from EDL933 in MG1655.

### 3.4 Effect of predation Stx phage production

**Figure 4:**
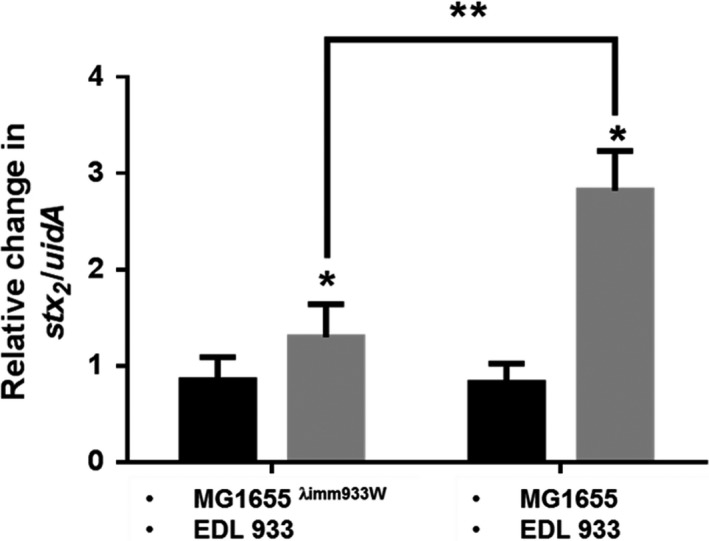
Facilitation in mixed bacterial population. Microcososms containing EDL933 and either MG1655^λimm933W^or MG1655 bacterial strains were incubated without (black bars) or with *Tetrahymena* (gray bars) at 30°C for 4 hr. Total DNA was isolated from these microcosms at time 0, and 4 hr. The amount of *stx2A* and *uidA* was quantified via qPCR and the relative change in *stx2A*:*uidA* ratio after 4 hr of co‐incubation is reported. The number of trials *n* ≥ 3, *p <* *.05, **p <* *.02

The Stx‐encoding phage in EDL933 carries only one copy of the *stx2A* gene in its genome (Plunkett, Rose, Durfee, & Blattner, 1999). Likewise, the *E. coli* strains also contain only one copy of β‐glucuronidase gene *uidA*(Sanjar et al., 2014). Thus, an increase in the ration of *stx2A*:*uidA* genes in a culture indicates the lytic growth of *stx* ^*+*^ phage. Therefore, we monitored phage production by determining the change in the ratio of *stx2A*:*uidA* alleles in the population by qPCR. For these experiments we measured the amount of phage produced in cocultures containing EDL933 and MG1655 or MG1655^λimm933^, in the absence or presence of predatory *Tetrahymena*. We found, that relative to control cocultures containing without *Tetrahymena*, phage production increased 1.5‐fold in the presence of *Tetrahymena* (Figure 4 left bars). Since the immune strain cannot be infected by Stx^+^ phage, any increase in *stx2A/uidA* ratio reflects the increase in Stx^+^ phage being released from EDL933 as a response to predation by *Tetrahymena*. By comparison, cocultures containing EDL933, MG1655, and *Tetrahymena* produce nearly threefold more phage than control cocultures containing only the two bacterial strains (Figure 4 right bars). Consistent with the idea that the a phage susceptible bacterial strain can be used as a surrogate to amplify the amount of exotoxin‐encoding phage produced by the population, a greater amount of phage is produced in the cocultures containing EDL933, MG1655, and *Tetrahymena* than those containing EDL933, MG1655^λimm933^, and *Tetrahymena*.

### 3.5 Effect of predation on lysogen formation

In addition to functioning as surrogates to increase exotoxin production via bacteriophage lytic growth, it is possible that the phage susceptible bacteria in a population may become lysogenized with the released exotoxin encoding phage, thereby allowing exponential growth of the phage population via subsequent replication of the bacterial lysogen. Phage‐mediated horizontal gene transfer would increase the probability that neighboring individuals bear the same allele, favoring investment to maintain the cooperative trait (Mc Ginty, Lehmann, Brown, & Rankin, 2013; Nogueira et al., 2009). To test this possibility, we used PCR to detect the formation of lysogens in cocultures consisting of a phage‐susceptible *E. coli* strain, an exotoxin‐encoding phage donor strain (EDL933), and predatory *Tetrahymena*. Since the presence of *Tetrahymena* causes induction of S*tx* ^+^ prophage (Lainhart et al., 2009), any prophage formed in our experimental regime will be susceptible to induction by this organism. To prevent this occurrence, we used a *rec*A mutant, phage susceptible strain (MG1655 *recA*), so any prophage formed in this strain cannot be subsequently induced. As controls, we examined lysogen formation in co‐cultures that lack the predator and in ones that contained a *rec*A mutant of the phage donor strain EDL933 (EDL933*recA*). The *recA* mutation prevents the Stx‐encoding prophage in EDL933*recA* from entering the lytic cycle. The phage susceptible MG1655*recA* and EDL933 bacterial strains express different antibiotic resistances allowing us to isolate only the recipient bacteria.

**Figure 5:**
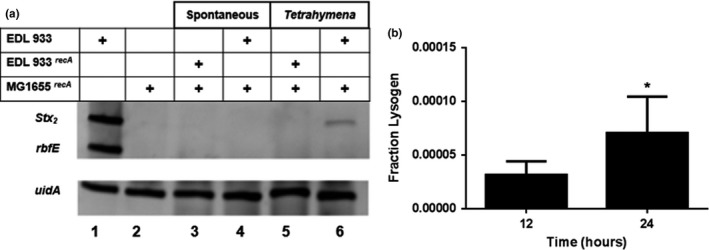
The bacteriophage transfer and lysogen formation. (a) Bacteriophage 933W transfer was detected using PCR. For these experiments, EDL933 (a 933W phage donor strain) or MG1655^*recA*^ (phage recipient bacterial strain) were separately cultured (lanes 1 and 2, respectively) or together in the absence (lane 4) or presence of *Tetrahymena* (lane 6) for 12 hr. Likewise, an EDL933 variant strain EDL933^*recA*^ was used to create a similar microcosm (lane 5). Following coincubation, MG1655^*recA*^ was separated from *Tetrahymena* and EDL933 or EDL933^*recA*^ as described in Experimental Procedures. The following genes: *stx2A* (933W phage specific), *rbfE* (EDL933 specific gene) and *uidA* (an internal DNA control) were amplified using PCR and the resulting products displayed on a PAGE gel. Lane 1 EDL933 alone; lane 2 MG1655 alone; lane 3 EDL933^*recA*^ and MG1655^*recA*^ incubated without *Tetrahymena;* lane 4 EDL933 and MG1655^*recA*^ incubated without *Tetrahymena,* lane 5 EDL933^*recA*^ and MG1655^*recA*^ incubated with *Tetrahymena;* lane 6 EDL933 and MG1655^*recA*^ incubated with *Tetrahymena,* (b) The frequency of phage transfer. A microcosm consisting of EDL933, MG1655^*recA*^, and *Tetrahymena* was constructed. After 12 and 24 hr of coincubation MG1655^*recA*^ was separated from *Tetrahymena* and EDL933 as described in Experimental Procedures. The amount of the following genes, *stx2*A (933W phage specific) and *uidA* was quantified via qPCR. The number of trials *n* ≥ 3, *‐*p *<* *.05

Formation of lysogens in the MG1655*rec*A recipient bacteria was detected by amplifying *Stx* ^*+*^phage specific gene *stx2A* (Plunkett et al., 1999) (Figure 5a, lane 1). To ensure there is no DNA carry over from the phage donor strain we monitored amplification of the EDL933 specific gene, *rbfE* (Desmarchelier et al., 1998) (Figure 5a, lanes 1, 3‐6). The *uidA* gene was amplified as an internal control (Figure 5a, lanes 1‐6). Our results show that Stx‐encoding prophage is found in MG1655*rec*A only in cocultures containing EDL933, the phage susceptible strain and *Tetrahymena* (Figure 5a, lane 6). In contrast, no Stx‐encoding prophage were found in MG1655*rec*A in the cocultures that contained EDL933 *recA*, a strain that is unable to produce phage (Figure 5a, lane 3, 5) or lacked the predator (Figure 5a, lane 3 and 4). Taken together, the findings in Figure 5 suggest that bacterivorous predators play an important role in facilitating transfer of Stx‐encoding prophage from a donor to a recipient bacterial strain in mixed bacterial populations.

In the MG1655*rec*A/MG1655*recA* ^933W^ population, each *stx2A* gene corresponds to one prophage (Plunkett et al., 1999) and each bacteria contains one *uidA* gene (Hayashi et al., 2006). Thus, we calculated the efficiency of lysogen formation by using qPCR to measure the ratio of *stx2A* :*uidA* genes in the population after coculturing phage susceptible MG1655*recA*, EDL933 and *Tetrahymena* for 12, and 24 hrs. At 12 hr, ~ 0.003% of the MG1655 were lysogenized with Stx‐encoding bacteriophage (Figure 5b), corresponding to >2,500 new lysogens/ml (see Experimental Procedures). The fraction of bacteria that are lysogenized with Stx‐encoding bacteriophage increases over time and is twofold higher at 24 hr. This finding indicates that new lysogens are being formed continuously under these conditions.

The relatively low level of MG1655*rec*A::933W lysogen formation seen in Figure 5b helps explain our somewhat surprising inability to detect new lysogens in MG1655*rec*A in cocultures between this strain and EDL933 in the absence of *Tetrahymena* (Figure 5a, lane 4). Our previous experiments indicate that that EDL933 undergoes spontaneous induction at low frequency under these conditions (Aijaz & Koudelka, 2017). Hence, the number of phage released, and thus their titer, is quite low. In contrast, confinement of EDL933 to the *Tetrahymena* phagosome significantly increases its spontaneous induction frequency (Aijaz & Koudelka, 2017). Hence, our inability to ‘see’ these new lysogens forming in cocultures containing MG1655*rec*A and EDL933 in the absence of *Tetrahymena* is seemingly due, in part, to a low phage titer that leads the formation of a small, and apparently undetectable number of new lyosgens.

## 4 DISCUSSION

Cooperative behavior is common in microbial populations. Many microbes secrete toxins that improve ecological conditions for the producer (Driscoll, Espinosa, Eldakar, & Hackett, 2013), acting either as antibiotics, impairing or eliminating microbial competitors and thereby reducing competition for limited resources (Chao & Levin, 1981; Driscoll et al., 2013; Pierson & Pierson, 2010) or as virulence factors that allow host invasion, paving the way for colonization and access to new/richer environments (Raymond, West, Griffin, & Bonsall, 2012). Previous studies showed that Shiga toxin (Stx), a bacteriophage‐encoded exotoxin, can kill bacterivorous single‐celled protist predators (Arnold & Koudelka, 2014; Lainhart et al., 2009; Steinberg & Grinstein, 2007; Stolfa & Koudelka, 2012).

In a bacterial population subjected to predation, only a small fraction of the Stx phage‐bearing bacteria are induced and produce toxin, a sacrifice that reduces predation and increases the survival of the population (Arnold & Koudelka, 2014; Stolfa & Koudelka, 2012). Thus, although Stx is an exotoxin, it improves the competitive fitness of bacterial populations that carry Stx‐encoding phage by a novel mechanism, that is, providing the bacterial host a means to defend against the predation by single‐celled protozoan bacterivorous predators. These observations argue that carriage of Stx‐encoding phage embodies a form of self‐destructive cooperation (Ackermann et al., 2008), with the fitness cost of this behavior being “paid” by death of the individual cell via and the fitness benefits accruing to the kin of sacrificed bacteria. This conclusion indicates that the phage‐borne Stx exotoxin behaves as a public good.

Our results indicate that the fitness benefits of Stx production are not restricted to the kin of the phage‐encoding bacteria (Figures 1, 2). Since many bacterial predators do not discriminate between bacterial food sources, prey bacteria in the population that do not encode Stx can cheat, selfishly reaping the fitness benefits of the antipredator activity of Stx released by STEC, while not investing in its production. Cheating may help explain why not all bacteria in a given population encode this exotoxin. Consistent with this idea, we found that survival of nontoxin encoding, cheating bacteria varies with the proportion of cheaters within the population (Figure 3). Thus, above a certain threshold level of Stx‐encoding bacteria, enough Stx is produced to reduce predation sufficiently so that the selfish nonproducers thrive in the presence of predators. More importantly, the increased survival of non‐Stx producers in cocultures with Stx producers is only seen in the presence of a predator that is sensitive to killing by Stx. This observation confirms that the antipredator activity of Stx serves as the public good.

Alternatively, if the population contains a below‐threshold level of Stx‐encoding bacteria, the selfish nonproducers do not benefit from the presence of Stx‐encoding bacteria (Figure 3). The negative dependence of cheater survival on cheater population size leads a dynamic equilibrium distribution of selfish and cooperating individuals in a population (Powers, 2011). Exotoxin‐encoding bacteria and phage are ubiquitously distributed in the environment, but their occurrence is sporadic or episodic and their environmental persistence varies (Casas et al., 2006; O'Brien et al., 1984). Since cheating and cooperation facilitate the coexistence of cooperative (Stx^+^) and selfish (immune, Stx^−^) individuals in the face of predation, these processes may, therefore, help explain the environmental occurrence and persistence of Stx‐encoding bacteria and phage.

The cheating by nontoxin‐encoding bacteria threatens the persistence of the cooperative behavior engendered by carriage of Stx‐encoding genes. Social evolution theory suggests that in the face of cheating, cooperation can be maintained when its benefits are directed preferentially to organisms carrying cooperation genes (Charnov, 1977; Hamilton, 1963, 1964, 1970; Smith, 1964). The problem of how to limit benefits to closely related organisms is particularly acute in cooperation that is mediated by a diffusible public good such as Stx. It is well established that the evolution and maintenance of cooperation is strongly influenced by population structure. Physical segregation of producers from nonproducers increases the likelihood that a diffusible public good secreted by cooperating cells will be utilized by other cooperators. However, organisms in our liquid cocultures used here are well‐mixed, a condition that mimics the conditions in which the target predator (*Tetrahymena*), producers and natural cheaters naturally interact.

How then might the cooperative behavior that is conferred by Stx‐encoding phage be reinforced in bacterial populations? Our data suggest two mechanisms. First, since Stx is produced only during phage lytic growth, bacterial death accompanies Stx release. Thus, Stx production is a form of self‐destructive cooperation, where a subset of individuals in a population die in order to help others (Ackermann et al., 2008). Since infectious phage are released along with toxin, the phage can function as a lethal anticompetitor tool against susceptible bacteria that do not carry them (Brown, Le Chat, De Paepe, & Taddei, 2006; Joo et al., 2006). The amplification makes phage carriers capable of efficiently invading well‐mixed populations, even when initially rare (Brown et al., 2006). In the specific case of Stx‐encoding bacteria, the phage susceptible bacteria are also apparently used as surrogates to amplify toxin production, thus further enhancing population survival (Figures 3, 4), a feature that has impacts on Stx production and toxicity in animals (Goswami, Chen, Xiaoli, Eaton, & Dudley, 2015). Second, since the Stx‐encoding phage are temperate, the released phage can lysogenize phage susceptible bacteria. Lysogeny directly increases the population of genetically similar cooperating individuals in a population.

These mechanisms explain the maintenance of cooperative behavior in populations consisting of Stx phage‐bearing and phage susceptible bacteria. However, what about more diverse communities formed by Stx phage‐bearing bacteria together with bacteria resistant to infection by the Stx‐encoding phage? In this case, our results suggest that in the presence of a predator, when Stx‐encoding bacteria are present at low levels, the predator will more likely encounter and consume the dominant members of the community, that is, the bacteria that do not encode Stx. Whereas when the Stx producers are present in higher density, the predator consumes them, but the predator is, in turn, killed by the Stx produced.

Genes encoding cooperative traits are often found in association with mobile genetic elements such as plasmids or transposons (Dimitriu et al., 2014). These mobile elements facilitate rapid horizontal gene transfer within and between bacterial lineages suggesting that they can quickly alter the cooperative social structure of a population. Horizontal gene transfer via transduction has traditionally been considered a rare event. However, recent studies have reported that transduction might occur at higher frequencies than previously thought (Evans et al., 2010; Kenzaka, Tani, & Nasu, 2010).

Protozoan predation increases the frequency of transfer, persistence, and spread of plasmids among bacteria (Cairns, Jalasvuori, Ojala, Brockhurst, & Hiltunen, 2016). Temperate bacteriophages have two lifecycles, lytic and lysogenic, so they have the potential to serve as a vessel in transferring genes encoding public goods across bacterial species. Our results suggest that protozoan predators, in particular those who feed indiscriminately, strongly enhance transfer of Stx‐encoding temperate phage from one susceptible host to another. Predation may enhance phage transfer by two nonmutually exclusive mechanisms. First, predation may increase the number of phage. We showed previously that consumption of bacteria by a protozoan predator stimulates prophage induction (Aijaz & Koudelka, 2017; Arnold & Koudelka, 2014; Lainhart et al., 2009; Stolfa & Koudelka, 2012).

Second, predation may increase contacts between phage and recipient bacteria. The filter‐like feeding behavior of *Tetrahymena* can result in the confinement of phage, its donor and recipient strains within a single phagosome, thereby enhancing the rate of phage infection in new hosts. The increased concentration of phage within the organelle would increase the multiplicity of infection, a condition that favors lysogen formation over lytic phage growth. Therefore, we believe that phages have contributed in the evolution of cooperative behavior of microorganisms.

We suggest that the constant struggle between predator and prey, cooperators and cheaters, shape the evolution of phages and if these phages carry toxins which can harm humans, as is the case with Stx2 encoding phage, then they shape the evolution of new human pathogens.

## CONFLICT OF INTEREST

None declared.
